# Demographic and clinical profile of patients with chronic rhinosinusitis in Saudi Arabia

**DOI:** 10.15537/smj.2023.44.4.20220947

**Published:** 2023-04

**Authors:** Rayan Alfallaj, Sultan bin Obaid, Hisham Almousa, Dawood Ismail, Saleh Mahjoub, Fatma Alanazy, Surayie Al Dousary, Saud Alromaih, Mohammad Aloulah, Abdulaziz Alrasheed, Ahmad S. Alroqi, Saad Alsaleh

**Affiliations:** *From the Department of Otolaryngology – Head And Neck Surgery Department (Alfallaj, Alanazy, Al Dousary, Alromaih, Aloulah, Alrasheed, Alroqi, Alsaleh) College of Medicine; and from the College of Medicine (bin Obaid, Almousa, Ismail, Mahjoub), King Saud University, Riyadh, Kingdom of Saudi Arabia.*

**Keywords:** chronic rhinosinusitis, nasal polyp, Samter’s triad, asthma, aspirin sensitivity

## Abstract

**Objectives:**

To determine the clinical features of patients with chronic rhinosinusitis at a tertiary hospital in Riyadh, Saudi Arabia.

**Methods:**

A cross-sectional study was carried out at King Abdulaziz University Hospital, Riyadh, Saudi Arabia. We enrolled 660 male and female participants with medical records indicating a history of chronic rhinosinusitis between 2021 and 2022. Quantitative and descriptive analyses of age, gender, nationality, presence of polyps, aspirin sensitivity, presence of urticaria, asthma, and allergies were performed.

**Results:**

Of the 660 enrolled patients, 60% (n=396) were male and 40% (n=264) were female. Additionally, 67.7% (447) had nasal polyps, 32% had a history of asthma, 10% had hypersensitivity to aspirin, 1.4% reported a history of urticaria, 9.7% reported allergies to medications, 7.9% reported food allergies, 26% reported multiple allergies, and 1.8% reported environmental allergies.

**Conclusion:**

Our study revealed the following: Samter’s triad was present in 6.9% of participants with chronic rhinosinusitis; the greatest prevalence of chronic rhinosinusitis with nasal polyps was observed among those older than 50 years. The prevalence of urticaria was not significantly different among groups; a higher rate of environmental allergies was observed among those with CRSwNP than among those without nasal polyps; and a higher prevalence of aspirin hypersensitivity was observed among those with CRSwNP than among non-polyps group.

**C**hronic rhinosinusitis (CRS) is characterized by an ongoing inflammatory process in the sinuses and nasal cavity. It is a prevalent medical issue with significant negative impact on the quality of life and cost of healthcare.^1^ Its prevalence is approximately 10% in Western populations.^2^ The criterion for the diagnosis of CRS is at least duration of 12 weeks of 2 or more of the following: nasal discharge or obstruction, facial pressure, and olfactory dysfunction.^2^ Chronic rhinosinusitis can be categorized as chronic rhinosinusitis with nasal polyps (CRSwNP) and chronic rhinosinusitis without nasal polyps (CRSsNP).^1^ Its diagnosis can be confirmed by nasal endoscopy or imaging techniques such as computed tomography.^2^ Although the majority of CRS cases occur in healthy individuals, a small percentage have different underlying etiological and pathological mechanisms, including genetic diseases, such as primary ciliary dyskinesia, cystic fibrosis, autoimmune disorders, or immunodeficiency.^2^ Numerous studies have emphasized the role of the association of asthma with CRS and its effects on the disease process and the clinical characteristics of CRS with and without asthma.^3^ The pathogenesis of CRS is attributed to abnormal mucociliary clearance, cell barrier abnormalities, and tissue reorganization.^4^ Genetic susceptibility and anatomic abnormalities also have roles in its pathogenesis.^5^ Exposure to allergens and pathogen infections are considered the most common factors responsible for the exacerbation of asthma, which may also have a role in initiating or worsening CRS.^1^ This study aimed to describe CRS and the demographics and clinical profiles of CRS patients at a tertiary hospital in Riyadh.

## Methods

We performed this cross-sectional study at King Abdulaziz University Hospital (KAUH), Riyadh, Saudi Arabia. We included male and female participants with medical records indicating a history of CRS from 2021 to 2022. Study approval was obtained by Ethical Committee of Research at College of Medicine, King Saud University (project number: E-21-6053). Quantitative (numbers and percentages) and descriptive analyses of age, gender, nationality, presence of polyps, aspirin sensitivity, presence of urticaria, asthma, and allergies were performed.

Raosoft software (Raosoft Sample Size Calculator. Raosoft, Inc., Seattle.) was used to identify the appropriate sample size for this analysis. The prevalence of chronic sinusitis in Saudi Arabia is estimated to be 25% among the total Saudi population of 36 million individuals, and the estimated prevalence of CRS is 9 million individuals; therefore, to achieve a 99% confidence interval, the required sample size was estimated to be 500 to 664 individuals (margin of error=5%). A simple random sampling technique was chosen for this study.

### Statistical analysis

The analysis of the data was performed using R (version 3.6.2). Variables were categorical or continuous. Categorical variables were described using numbers and percentages, whereas the continuous variables were represented by mean and standard deviation. Assessment of the association between categorical variables were carried out using Chi-square analysis of independence. Spearman’s correlation was used to determine correlations among variables. Hypothesis testing with a 5% level of significance was performed.

## Results

A total of 660 patients with CRS were included. Of these, 60% (n=396) were male and 40% (n=264) were female. One-third of the participants were older than 50 years of age. One-third of the participants were 35 years of age or younger (30.5%). The remaining one-third of participants were 36 to 50 years of age (38.4%). The average age of the participants was 40.47 years (standard deviation, ±9.4 years). Most of the participants were Saudi (96.2%). Of the included 660 patients, 447 (67.7%) had nasal polyps, 44 (9.8%) had aspirin-exacerbated respiratory disease, 26 (5.8%) had AFRS, 32% had a history of asthma, 10% had hypersensitivity to aspirin, and 1.4% had a history of urticaria (Table 1). Furthermore, 9.7% of patients reported allergies to medications, 7.9% reported food allergies, 26% reported multiple allergies, and 1.8% reported environmental allergies (Figure 2). Factors associated with CRS without nasal polyps and distributions according to gender and nationality were not statistically different among those with and without nasal polyps (*p*=0.709) (Table 1). Age distributions of patients with and without nasal polyps were statistically significantly different (*p*<0.001). The prevalence of polyps was highest among patients older than 50 years of age, and it was lowest among those 31 to 35 years of age. Prevalence of CRSwNP is significantly higher (89.4%) than CRSsNP (10.6%) in the aspirin hypersensitive population in this study. The prevalence of urticaria was not significantly different between groups. The prevalence of nasal polyps was lower among patients with no allergies than among those with food, environmental, or medication allergies (*p*<0.001). Nasal polyps were also more prevalent among patients with asthma (80.6%) than among those without asthma (61.7%) (*p*<0.001). Gender was significantly associated with aspirin-exacerbated respiratory disease (*p*=0.003). The prevalence of aspirin-exacerbated respiratory disease was higher among females (10.6%) than among males (4.5%). There was a statistically significant positive association between age and nasal polyps (r=0.163; *p*<0.001). Positive associations were also observed between age and asthma (r=0.163; *p*<0.001), hypersensitivity to aspirin (r=0.154; *p*<0.001), and Samter’s triad (r=0.154; *p*<0.001).

**Figure 1:**
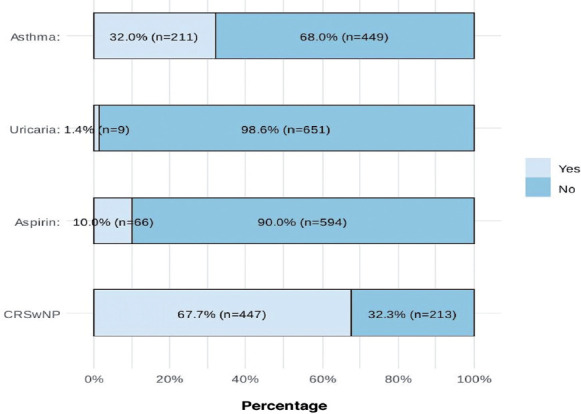
- History of nasal polyps and allergy in the included respondents.

**Figure 2:**
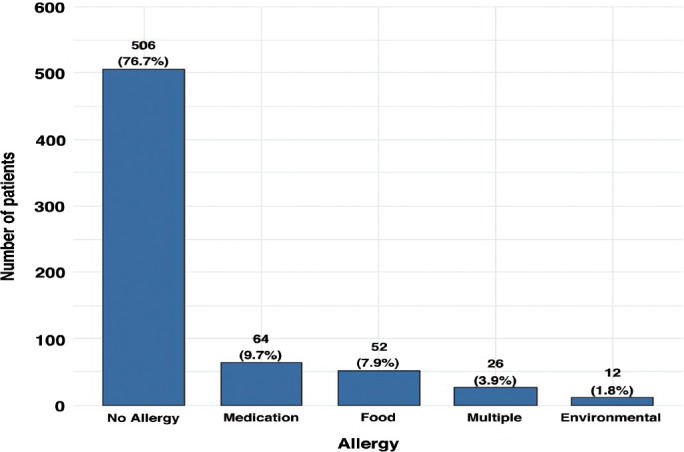
- History of nasal polyps and allergy in the included respondents.

**Table 1:** - Factors associated with CRS groups.

Variable	CRSwNP	CRSsNP	*P*-value
***Gender***			0.709
Female: 264 (40.0)	68.6))181	83 (31.4)	
Male: 396 (60.0)	266 (67.2)	130 (32.8)	
***Age***			<0.001**
<20:10	1 (10.0)	9 (90.0)	
20-25:48	31 (64.6)	17 (35.4)	
26-30:66	40 (60.6)	26 (39.5)	
31-35:77	39 (50.6)	38 (49.4)	
36-40:100	62 (62.0)	38 (38.0)	
41-45:79	61 (77.2)	18 (22.78)	
46-50:75	57 (76.0)	18 (24.0)	
>50:205	156 (76.1)	49 (23.9)	
***Nationality***			0.400
Saudi: 635	432 (68.0)	203 (31.96)	
Non-Saudi: 25	15 (60.0)	10 (40.0)	
***Aspirin sensitivity***			<0.001**
Yes:66	59 (89.4)	7 (10.6)	
No:594	388 (65.3)	206 (34.7)	
***Urticaria***			0.432
Yes:9	5 (55.6)	4 (44.4)	
No:651	442 (67.9)	209 (32.1)	
***Asthma***			<0.001**
Yes: 211	170 (80.6)	41 (19.4)	
No: 449	277 (61.7)	172 (38.3)	
***Allergy***			<0.001**
No allergy: 506	320 (63.2)	186 (36.8)	
Food allergy: 52	41 (78.8)	11 (21.2)	
Environmental: 12 allergy	10 (83.3)	2 (16.7)	
Medication allergy: 64	55 (85.9)	9 (14.1)	
Multiple allergy: 26	21 (80.8)	5 (19.2)	
***AERD***			<0.001**
Yes	44 (95.7)	2 (4.3)	
No	403 (65.6)	211 (34.4)	

Values are presented as number and percentages (%). Analysis was performed using Chi-square test of independence. CRwNP: chronic sinusitis with nasal polyps, CRS: chronic rhinosinusitis, AERD: aspirin exacerbated respiratory disease, **Significant at *p*<0.05

## Discussion

Our study investigated the clinical profiles and demographics of CRS patients in Saudi Arabia. The results revealed that the prevalence of nasal polyps was 67.7%. Forty-four (9.8%) had aspirin-exacerbated respiratory disease, 26 (5.8%) had AFRS, 32% had asthma, 10% had hypersensitivity to aspirin, and 1.4% had urticaria. A large national study conducted in the United Kingdom found that among 1249 participants with CRS, 553 (44.2%) had CRS without nasal polyps, 651 (52.1%) had CRS with nasal polyps, 45 (3.6%) had AFRS, and 9.6% had aspirin intolerance.^6^ A study performed in Europe by Langdon and Mullol^7^ found that asthma was associated with 20% to 60% of cases of CRS with nasal polyps. Moreover, our study findings revealed that Samter’s triad was present in 6.9% of participants with CRS, which was higher than the prevalence among the European adult population (1%).^7^ The prevalence of CRS with nasal polyps is 2.5% to 2.6% in the general population in South Korea, which is greater than that reported for the general population in the USA at 1.1%. Moreover, the prevalence of NP is 6.1% to 31% in the CRS population in the United States and 24% in the CRS population in Denmark.^8^ A study performed in Korea showed that CRS with nasal polyps was more prevalent among males, with the greatest prevalence among those 60 to 69 years of age. These results are consistent with those of our study, which showed the greatest prevalence among those older than 50 years of age; however, in our study, gender was not statistically different among groups.^8^ Stevens et al^9^ reported the prevalence of isolated CRS with nasal polyps was 43% among the adult population, whereas asthma was found in 39% of those with polyps and Samter’s triad was found in 16% of those with polyps in the United States. The relationship between chronic urticaria and nasal polyps remains unclear in the literature, which is consistent with our finding that the prevalence of urticaria was not significantly different among groups.^10^ In our study, a higher rate of environmental allergies was observed among those with CRS with nasal polyps than among those with CRS without nasal polyps, thus supporting the findings of Pumhirun et al.^11^ Pumhirun et al^11^ performed a skin prick test to determine environmental allergies among those with CRS with nasal polyps and a control group and found a higher rate of sensitivity among those with CRS with nasal polyps. A lower prevalence of nasal polyps was found among patients with no allergies than among those with food allergies, which is consistent with the findings of Pang et al^2^ who found that those with polyps had more positive food allergen intradermal test results. A study performed in Iran revealed that the prevalence of aspirin hypersensitivity was 43.8% among patients with CRS with nasal polyps, which was higher than that among the controls.^13^ However, our study showed a prevalence of aspirin hypersensitivity (13%) among those with CRS with nasal polyps. We found that the prevalence of Samter’s triad was higher among females (10.6%) than among males (4.54%); this difference was significant. Furthermore, this result was consistent with that of Li et al,^14^ who found a female-to-male ratio of 3:2 for the presence of Samter’s triad among those in their third or fourth decade of life; however, our findings associated with age and nationality were not significantly associated with Samter’s triad.

### Study limitation

The retrospective design of this study and the small sample size were the main limitations of this study. Type of management, recurrence rate, and the need for revision surgery was not addressed and future research in that regards is needed. However, this study remains one of the few to describe the characteristics of CRS in Saudi Arabia and paves the way for future research on a national level.

In conclusion, among patients with CRS in Saudi Arabia, 67.7% had nasal polyps, 32% had associated asthma, 10% had aspirin sensitivity, and 1.4% had a history of urticaria. Our study revealed that Samter’s triad was present in 6.9% of participants with CRS. It also revealed that the greatest prevalence of CRS with nasal polyps was observed among those above the age of 50 years. The prevalence of urticaria was not significantly different among groups. A higher rate of environmental allergies was observed among those with CRSwNP than among those without nasal polyps. Finally, a higher prevalence of aspirin hypersensitivity was observed among those with CRS with nasal polyps than among those with CRS without nasal polyps. Additional studies are required to further evaluate these patients so that the best management practices can be performed for this prevalent disease.
